# Qualitative Description of Global Health Nursing Competencies by Nursing Faculty in Africa and the Americas

**DOI:** 10.1590/1518-8345.0772.2697

**Published:** 2016-06-07

**Authors:** Lynda Wilson, Laura Moran, Rosa Zarate, Nicole Warren, Carla Aparecida Arena Ventura, Irene Tamí-Maury, Isabel Amélia Costa Mendes

**Affiliations:** 1 PhD, Professor Emerita, School of Nursing, University of Alabama at Birmingham, AL, United States.; 2 EdD, EdM, Full Professor, Escuela Nacional de Enfermería y Obstetricia, Universidad Nacional Autónoma de México, Mexico City, Mexico.; 3 PhD, Professor, Escuela Nacional de Enfermería y Obstetricia, Universidad Nacional Autónoma de México, Mexico City, Mexico.; 4 PhD, Assistant Professor, School of Nursing, Johns Hopkins University, Baltimore, United States.; 5 PhD, Associate Professor, Escola de Enfermagem de Ribeirão Preto, Universidade de São Paulo, PAHO/WHO Collaborating Centre for Nursing Research Development, Ribeirão Preto, SP, Brazil.; 6 Assistant Professor, Department of Behavioral Science, University of Texas, MD Anderson Cancer Center, Houston, TX, United States.; 7 PhD, Full Professor, Escola de Enfermagem de Ribeirão Preto, Universidade de São Paulo, PAHO/WHO Collaborating Centre for Nursing Research Development, Ribeirão Preto, SP, Brazil.

**Keywords:** Global Health, Global Nursing, Competencies, Qualitative Analysis

## Abstract

**Objective:**

to analyze qualitative comments from four surveys asking nursing faculty to rate the importance of 30 global health competencies for undergraduate nursing programs.

**Method:**

qualitative descriptive study that included 591 individuals who responded to the survey in English (49 from Africa and 542 from the Americas), 163 who responded to the survey in Spanish (all from Latin America), and 222 Brazilian faculty who responded to the survey in Portuguese. Qualitative comments were recorded at the end of the surveys by 175 respondents to the English survey, 75 to the Spanish survey, and 70 to the Portuguese survey. Qualitative description and a committee approach guided data analysis.

**Results:**

ten new categories of global health competencies emerged from the analysis. Faculty also demonstrated concern about how and when these competencies could be integrated into nursing curricula.

**Conclusion:**

the additional categories should be considered for addition to the previously identified global health competencies. These, in addition to the guidance about integration into existing curricula, can be used to guide refinement of the original list of global health competencies. Further research is needed to seek consensus about these competencies and to develop recommendations and standards to guide nursing curriculum development.

## Introduction

The impact of globalization on the social, political, economic, and environmental issues that affect community and individual health around the world is unquestionable. Health problems have crossed borders due to the increase of global travel and migration, as well as displacements caused by war, violence, and natural disasters. The recent Ebola epidemic is but one example of a global health problem that transcends national borders. There are calls for increased multinational partnerships to address these problems in the post-2015 United Nations development agenda^1^. There is growing recognition of the need for nurses and other health professionals to have competencies to address global health problems^2^ ^-^ ^3^. 

**Figure 1:**
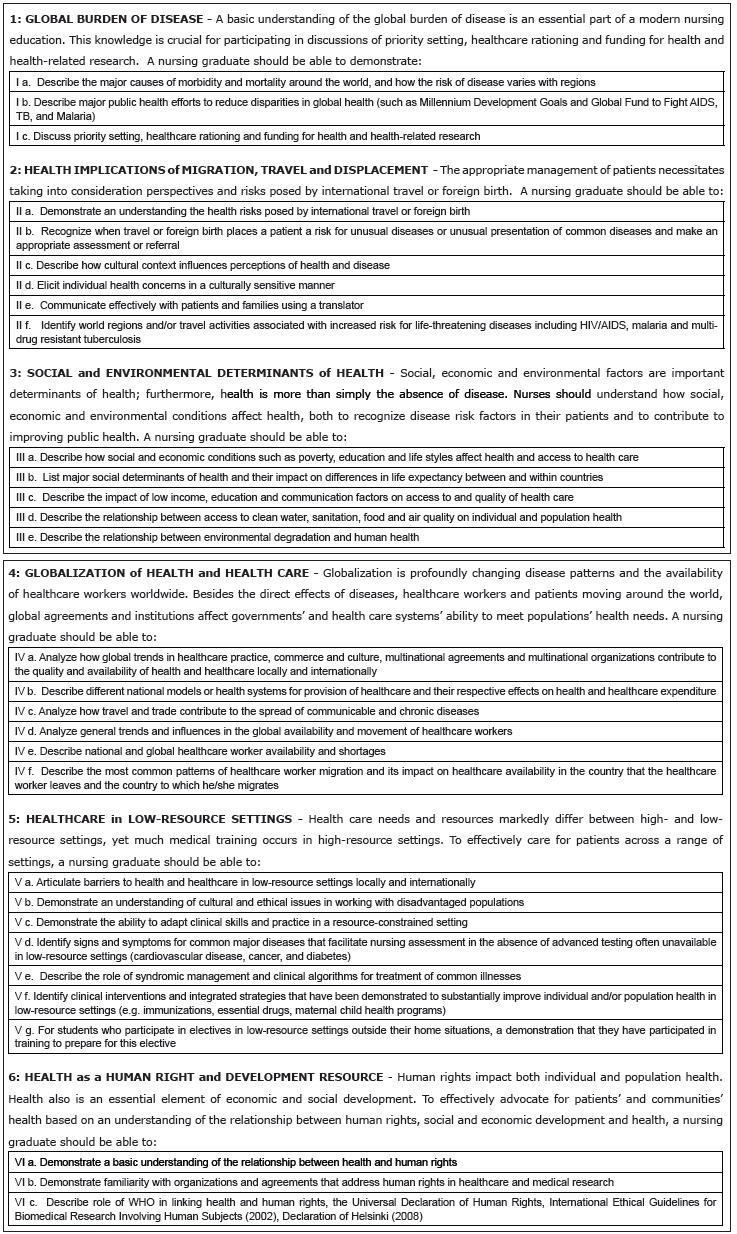
List of Global Health Competencies Included in Original Studies^2^ ^-^ ^3^

Several recent studies have been conducted to identify competencies related to global health that might be integrated into nursing curricula. In the first study, Wilson and colleagues adapted a list of global health competencies that had been developed for medical students, and sent the list via email to nursing faculty in the United States, Canada, Latin America, and Caribbean countries, asking them to complete an online survey ranking the extent to which they believed that each of the 30 competencies should be addressed in undergraduate nursing programs^3^. The 30 competencies are divided into six subscales: (a) Global Burden of Disease, (b) Health Implications of Travel and Displacement, (c) Social and Environmental Determinants of Health, (d) Globalization of Health and Health Care, (e) Health Care in Low Resource Settings, and (f) Health Care as a Human Right and Development Resource. These competencies are available in the previously published paper^2^ and in Figure 1). Respondents were asked to apply the following definition of global health^4^ when responding to the survey or when identifying additional competencies that should be included in nursing curricula: "an area for study, research, and practice that places a priority on improving health and achieving equity in health for all people worldwide. Global health emphasizes *transnational health issues, determinants, and solutions;* involves many disciplines within and beyond the health sciences and promotes interdisciplinary collaboration; and is a synthesis of population based prevention with individual-level clinical care."^4^

A total of 593 nursing faculty members responded to the original survey^3^ (542 English responses and 51 Spanish responses), and the quantitative findings indicated general agreement that the competencies should be integrated into undergraduate nursing curricula. In a follow-up survey sent to all nursing schools in Brazil, 222 Brazilian faculty also indicated strong agreement that the global health competencies should be integrated into nursing curricula^2^. An additional 112 responses to the Spanish survey were received when the survey was repeated in Spanish-speaking Latin American countries with collaboration of the Latin American Association of Nursing Schools (ALADEFE) and the Nursing School of the UNAM. An additional 49 English surveys were received when the survey was replicated in Africa. Quantitative findings from those surveys were consistent with the findings from the initial surveys suggesting support for inclusion of the competencies in nursing curricula^5^. In addition to rating the relevance of each competency for undergraduate nursing education, respondents provided open-ended qualitative responses to a question asking them to identify additional competencies or provide comments about the competencies. Although the publication describing the initial survey results provided a brief overview of the themes from these qualitative comments, the present paper provides a more in-depth analysis of the qualitative responses to all of the surveys conducted to date. The purpose of this paper is to present the themes that emerged from the qualitative comments provided in the global competency surveys in order to identify additional competencies that were not included in the original list of global health competencies for nurses, and identify issues related to integration of the competencies in nursing curricula. The specific research questions guiding the study were: (a) What are nursing faculty members' perceptions about additional essential global health competencies for undergraduate nursing students, in addition to those included in the original studies^2^ ^-^ ^3^ and (b) What recommendations do faculty have about integration of global health competencies into undergraduate nursing programs?

## Methods 

This cross-sectional, qualitative descriptive study relied on a non-probability snowball sampling procedure to recruit nursing faculty members from the United States, Canada, Caribbean countries, Latin America and Africa. Information and links to survey were distributed via email through a variety of contacts in the regions, and email recipients were asked to disseminate the survey to nursing faculty members who taught in schools of nursing that offered baccalaureate nursing programs. Further information on the sampling methods that were used and demographic characteristics of the respondents are described elsewhere^(2- 3)^. The original studies were approved by the research ethics committees of the University of Alabama at Birmingham, Johns Hopkins University, National Autonomous University of Mexico, and the University of Sao Paulo School of Nursing of Ribeirao Preto, Brazil. The introductory paragraph of the online survey informed participants that their completion of the survey implied their consent to participate in the study. All responses were anonymous. The sample for the original studies included 604 individuals who responded to the English survey (62 from Africa and 582 from the Americas or Caribbean countries), 163 who responded to the Spanish survey (all from Latin America), and 222 Brazilian faculty who responded to the Portuguese survey. Qualitative comments about additional competencies that should be considered or about integration of the competencies into nursing curricula were recorded at the end of the surveys by 175 English, 75 Spanish, and 70 Portuguese-speaking respondents. 

The goal of our qualitative analysis was to identify and describe competencies that were not included in the original list of global health competencies for nurses^2^ ^-^ ^3^. Our data set included comments in English, Spanish and Portuguese. There are multiple challenges to high-quality translation in survey research and little information exists about how to ensure rigorous translation of qualitative data^6^ ^-^ ^7^. Therefore, we used qualitative description and a committee approach to translation to guide our data analysis. Qualitative description provides a descriptive summary of the data collected without an attempt to reinterpret the participants' comments^8^. This is in contrast to other qualitative methodologies, such as phenomenology or grounded theory that create theory and/or new understandings from the data. Because our qualitative data were limited to brief open-ended statements, qualitative description permitted us to describe and organize our findings without an attempt to re-interpret participants' comments. A committee approach is considered preferable to the work of a single translator^6^ ^-^ ^7^, and involves multiple translations of the same document that are later discussed as a group. The final version is agreed upon by consensus. The committee approach guided both the translation of the comments and the analysis of the comments to identify themes and categories for coding. 

To begin, each researcher individually reviewed the qualitative responses in her native language data-set. Then, we broke into teams by native language: two native English speakers, three native Spanish speakers, and two native Portuguese speakers. Team members with the same native language compared coding ideas and came to a consensus on the initial codes for each language. When a preliminary coding scheme had been agreed on within language groups, the entire team participated in online virtual meetings to review the codes across groups. These discussions were conducted in both Spanish and English with simultaneous translation by the first author. In addition, because native Spanish and Portuguese speakers on the team also spoke English, additional clarifications could be made. Through an iterative process, we applied developing coding definitions to the data and came to consensus about the final coding categories. The process was repeated until all research team members were in agreement about how the codes were being applied to the data. Throughout the analysis, the number of times an idea was mentioned was recorded and we report those frequencies here. Although quantitizing qualitative data can be controversial, it does help identify patterns which was, for our purposes, essential to demonstrating consensus^9^. 

A number of strategies have been identified to enhance rigor in qualitative analyses, and several of those strategies were incorporated in the analysis reported here^10^. The committee approach that was used for the translation and analysis of the comments helped to enhance the rigor of the analysis by promoting investigator triangulation in which two or more researchers make coding and analytic decisions to "reduce the possibility of biased decisions and idiosyncratic interpretations of the data"^10^ ^).^ The committee approach also supports the confirmabiity of the analysis which has been described as the "potential for congruence between two or more independent people about the data's accuracy, relevance, or meaning"^10^. Another component of rigor in qualitative analysis, transferability, refers to "the extent to which the findings can be transferred to or have applicability in other settings or groups"^10^ ^).^ In this study, the inclusion of data from multiple respondents representing diverse countries and languages, and the sharing of specific quotes and descriptions of comments that were coded in the different categories can help readers reach conclusions about whether the analytic interpretations and conclusions can be transferred to their own unique settings. 

Data that reflected existing global health competencies were not included in our analysis because we were trying to identify competencies that had not already been represented in the six subscales. Where data seemed related to but distinct from a competency already described in the six subscales, we differentiated how it was unique in our analysis. 

## Results 

The qualitative responses were coded into ten categories that related to additional competencies that should be added to the original list of 30 global health competencies, and four categories that reflected comments related to integrating these competencies into nursing curricula. A summary of the numbers of comments coded in each category, by the language of the respondent, is available upon request. A total of 30 qualitative comments reflected content that the researchers determined was already included in the original list of 30 competencies, and those comments are not included in the findings presented here. This section presents findings related to the qualitative comments that were coded in each category, presented in the order of the frequency of responses coded in each category. Although some of the categories address concepts that might already be included in nursing curricula, these concepts reflect components of the definition of global health that was used to guide the initial studies to identify global health nursing competencies, and thus they are included in the present report^4^ ^).^ Examples of comments for each category are included in italics for illustration purposes.

### Categories Reflecting Additional Competencies to be Developed

#### Culturally Competent, Humanistic, and Holistic Care

This category reflected content related to provision of culturally competent, holistic, equitable, ethical, and humanistic care with dignity. A total of 42 comments were coded in this category (23 English, 11 Spanish, and 8 Portuguese). There were two items in the original list of competencies related to providing culturally sensitive care (Item IIc, "Describe how cultural context influences perceptions of health and disease," and IId, "Elicit individual health concerns in a culturally sensitive manner"). The researchers determined that the comments coded in this category reflected a need for additional competencies that were more comprehensive than those in the original list. Comments coded in this category reflected the importance of *cultural competence and humility, understanding of transcultural nursing theories, and providing holistic and humanistic care*. 

#### Prevention, Health Promotion, and Primary Health Care

This category included 30 comments (14 English, 11 Spanish, and 5 Portuguese) related to prevention of illness, promotion of health (including health education and environmental health), and primary health care. Although the fifth category in the original competency list focused on "Healthcare in Low-Resource Settings", there were no competencies specific to health promotion or primary health care in the original list. An example of a comment that was coded in this category is: *Emphasis on Primary Health Care as an alternative to medical model in a resource-restrained setting.*

#### Multidisciplinary Work, Teamwork

Content coded in this category related to working in interprofessional or multidisciplinary teams or with partners/stakeholders in global health. There were 29 comments coded in this category (9 English, 2 Spanish, and 18 Portuguese). An example of a comment in this category was: *Ability to work in multi-disciplinary (physicians, faith healers, shaman, medicine people, physiotherapists, child development workers, families, occupational therapists, water analysts, social workers, activists, NGOs) and complementary settings. For example, competency - undergraduate nursing students learn to work with countries un-regulated health care workers, complementary team members (lay midwives, population health workers, community health representatives).*

#### Communication

This category reflected content related to communication competencies including communicating with patients, communities, other professionals, and communicating in a second language. A total of 22 comments were coded in this category (8 English, 10 Spanish, and 4 Portuguese). These comments reflected the importance of *knowing a second language, as well as ability to communicate with other professionals, patients, and family members*. 

#### Professional Nursing Issues in Diverse Settings

This category included 22 comments (11 English, 2 Spanish, and 9 Portuguese) related to the identification of legal, political, economic and educational issues relative to the preparation and work of nurses in the health sector in diverse national and international settings. Examples of comments that were coded in this category are: (a) *Know formation and work conditions of nursing in the world; and (b) Discuss the role and impact of nurses globally.*

#### Policy/Politics and Historical Context

This category included content related to historical and political factors influencing global health. A total of 21 comments were coded in this category (10 English, 5 Spanish, and 6 Portuguese). Examples of comments that were coded in this category include: (a) "*Recognize and articulate the implications of historic global inter-relationships between colonization and health equity"; and (b) "To be able to articulate the role of policy development and enactment in addressing health inequities."*

#### War, Disaster, Pandemics, Terrorism, and Displacement

A total of 17 comments (7 English, 3 Spanish, and 7 Portuguese) were coded in this category that included content related to adapting to uncertain situations to offer optimal health care in case of disaster, war, pandemics, displacement and terrorism. An example of a comment coded in this category is: *I think (it is) important to understand disaster planning and response, as well pandemic planning and response. Some knowledge of people being displaced, refugees, war and conflict and the impact particularly on mental health.*

#### Vulnerable Populations

Fourteen comments (9 English, 2 Spanish, and 3 Portuguese) were coded in this category that included content related to vulnerable populations such as individuals with disabilities, mental health problems, indigenous groups, individuals living in poverty, or women and children. Although one of the original competencies in the category focused on Healthcare in Low Resource Settings focused on "demonstrating an understanding of cultural and ethical issues in dealing with disadvantaged populations," none of the original competencies focused specifically on vulnerable populations. Examples of comments coded in this category included: (a) *Address violence to women and children, violence to pregnant women, lack of contraception and fertility education; and (b) Skills for working with indigenous populations and immigrants from countries which border with Brazil.*

#### Program Development, Planning, and Evaluation

A total of 13 comments (5 English and 8 Portuguese) were coded in this category which included content related to *evidence-based practice, planning, implementation, and evaluation of nursing interventions or health programs*. 

#### Leadership, Management, and Advocacy

This category included content related to leadership, management, and advocacy in global health. A total of 12 comments were coded in this category (6 English, 2 Spanish, and 4 Portuguese). An example of a comment coded in this category is: *Development of related public policy , management and organizational leadership skills in the area of ​​health .*

### Codes Related to Integrating the Competencies into Nursing Curricula

#### Leveling the Competencies

Five respondents (4 to the English and 1 to the Portuguese survey) wrote comments related to the need to identify different levels of competencies for different levels of nursing programs. Examples of these comments are: (a) *Most competencies described should be taught to all nurses; however, a few seem advanced for undergraduates - all graduate nurses should know all these competencies; and (b) These vary dramatically depending on the program. For undergraduate nurses, the most important competencies fall around their ability to identify, describe, and assess the complexities of social determinants of health, health inequities, and disparities. There should be separate competencies for undergraduate students' general preparation and for those who might participate in some form of international and/or global health exchange/placement.*

#### Overcrowding of the Nursing Curriculum

Five English-language comments were related to the challenges of adding global health content due to overcrowding of the nursing curriculum. Examples include: (a) *Well, it's hard to disagree with any of those goals but then again one wonders if all of this is Undergraduate content and how much more should be squeezed into already tight curricula; and (b) Global health should be an advanced practice major or perhaps we should just conclude that it will take six years to get a baccalaureate in nursing degree. None of the topics stated are unimportant and many are addressed already in BSN curricula. However, my negative responses were to those questions that while important and I would actually agree that a nurse should know and understand are unrealistic in terms of the accreditation constraints on what needs to be included in an undergraduate nursing curriculum. It is overwhelming already in terms of what has to be in an UG nursing curriculum. Perhaps what is more important is a discussion on what the future of nursing will be and what will be the "care" of nursing. In today's nursing schools I think including all the content r/t global health is beyond the pale!*

#### Relevance of Global Health to Nursing Education

Sixteen comments (14 from respondents to the English survey , 1 from a Spanish respondent, and 1 from a respondent to the Portuguese survey) addressed the perceived relevance of global health to nursing education. Five of the 15 comments reflected a "negative" perception of the view that adding global health competencies is relevant for undergraduate nursing education. For example: *Give me a break. Undergraduate students are still trying to figure out how to survive in a med/surg setting now they need to be competent in resource limited settings too. Give them a general education and basic professional education and they will figure it out.*

Ten of the 15 comments coded in this category reflected a perception that adding global health competencies was relevant for undergraduate nursing education. For example: *Nurses in all facets of healthcare do not operate in a vacuum. The international climate of healthcare impacts the practice of us all. Nurses at all levels need to be alert to the circumstances affecting healthcare consumers. As the world becomes smaller, through globalization, immigration, etc. the problems and concerns are not 'other there' or 'those people'.*

#### Strategies to Teach Global Health

Nine respondents (6 to the English survey and 3 to the Portuguese survey) wrote comments about specific strategies that could be used to teach global health content. For example: *I would encourage all programs to provide experiences in settings very different from what they are familiar with; and (b) International work should be a required part of community health.*

## Discussion

Traditional academic and technical competencies are no longer sufficient as nurses face increasingly higher personal and professional requirements in a globalized world. In this study, participants shared multiple examples of additional competencies that could be used to refine the list of 30 global health competencies that were included in the original surveys.

The authors acknowledge that the initial list of 30 competencies, as well as the categories of competencies that were identified from the qualitative responses may include competencies that are already included in nursing curricula. There is a need for further research to determine the extent to which the proposed competencies are already being included in nursing curricula. Such studies would help to identify additional global health competencies that should be integrated to supplement and complement existing curricula. 

Although themes related to specific content areas, such as War and Disasters and Vulnerable Populations, draw attention to important topics, it is noteworthy that several themes presented here are not content-specific but rather speak to an attitude or approach to providing care and/or working with others to do so. For example, the Culturally Competent, Humanistic, and Holistic Care theme reorients the nurse to his/her patient's perspective and unique needs. Similarly, the themes related to Communication and Multidisciplinary Work reinforce that the manner in which we work is what will make us effective in a global setting, not necessarily specific disease or intervention-related details. 

The theme related to *Culturally Competent, Humanistic and Holistic Care* was the theme identified most frequently. Although there were two items related to cultural competency in the original list of global health competencies, the qualitative comments identified additional concepts that should be incorporated such as *cultural competence and humility, understanding of transcultural nursing theories, and providing holistic and humanistic care.* Others have identified these concepts as important components of humanistic and holistic nursing care^11^. Cultural competence is already integrated into nursing curricula in many countries. In the United States, for example, the "Essentials" for baccalaureate nursing education" document includes the application of "knowledge of social and cultural factors to the care of diverse populations"^12^ ^).^ The International Council of Nursing has also published a position paper outlining competencies for culturally competent care^13^. Educators are challenged to facilitate students' understanding about how diversity characterizes and shapes human experiences^14^, and to help them understand others' perspectives and to communicate with and care for in culturally diverse contexts^15^.

The theme related to *Prevention, Health Promotion, and Primary Health Care* was the second most frequently identified theme. There were no specific competencies related to these concepts in the original list. The current emphasis on primary health care and universal health coverage suggests that these competencies should be integrated into any list of global health nursing competencies^(16- 17)^.

The third most frequently mentioned theme was related to *Multidisciplinary Work and Teamwork.* This theme is consistent with recent calls for the transformation of health professional education in the 21^st^ century and with the need for interprofessional education and collaborative practice^18^ ^-^ ^19^.

The themes related to *Communication* and *Understanding Professional Nursing Issues in Diverse Settings* were mentioned with equal frequency. Communication competencies are essential in all aspects of nursing, and particularly in a global and cross-cultural context^20^ ^-^ ^21^. Although most nursing educational programs already emphasize the importance of communication, a unique theme identified in the present study was the importance of acquiring skills in a second language. No global standards or guidelines were identified specifically related to foreign language requirements for nursing students, although there is growing recognition of the importance of knowing a second language in our increasingly globalized world^22^. The need for competency in working with a translator (or interpreter) was included as Competency IIe in the original list of competencies, and is an important communication competency for all global nurses. Although three of original competencies in the original list focused on analyzing trends in the availability and movement of healthcare workers and migration of healthcare workers, the original competencies did not focus specifically on professional nursing issues, suggesting the need to incorporate these competencies in a comprehensive list of nursing global health competencies.

Comments related to understanding *Policy, Politics, and Historical Context* were included in the list of additional competencies to consider, even though the original competency list included items related to analyzing how multinational organizations contribute to health. The original list of competencies, however, did not include competencies specifically related to an understanding of political and historical factors influencing health. In order to build global health competencies, nurses must understand legal, political, economic and educational issues related to nursing and health care in different settings^15^. 

Competencies related to dealing with *War, Disasters, Pandemics, Terrorism and Displacement* were not included in the original list of global health competencies. There have been previous initiatives to identify competencies related to disaster preparedness for nurses and other health professionals that might be reviewed and incorporated into a comprehensive list of global health competencies^23^ ^-^ ^24^.

Respondents suggested the importance of competencies related to *Program Development, Planning and Evaluation*. The Association of Schools of Public Health identified competencies related to program planning as essential for Master's in Public Health programs focused on global health^25^. Although undergraduate nursing students may not need advanced program planning/evaluation skills, a basic understanding of these skills would strengthen nurses' contributions to complex global health issues.

Respondents also identified the importance of competencies related to *Leadership, Management and Advocacy in Global Health and Working with Vulnerable Populations*. Although leadership, management and advocacy competencies are included in most nursing curricula, the fact that the respondents identified these suggests that these competencies may be particularly important in global health work. Because of the significant impact of social and environmental determinants on individual and population health, globally competent nurses must also have an understanding of care for vulnerable populations.

Four additional themes were identified that related to the integration of global health competencies into nursing curricula: *Leveling the Competencies, Overcrowding of the Nursing Curriculum, Relevance of Global Health to Nursing Education and Strategies to Teach Global Health*. Respondents expressed the need to identify different levels of global health competencies for diverse nursing programs, and several expressed concerns about adding additional global health competencies to already overcrowded nursing curricula. It is essential to carefully consider these concerns in future research and in the identification of strategies to engage faculty in incorporating global health competencies into nursing curricula. 

## Conclusion

Although the findings from these qualitative analyses can contribute to refinement of a comprehensive list of global health competencies, there is a need for further research to seek consensus about these competencies and to develop recommendations and standards to guide nursing curriculum development. A strength of the analysis reported here was the collection and analysis of qualitative responses from 295 nursing faculty in Africa, Latin America, and North America. Although the specific strategies for operationalizing the competencies may differ among nursing programs in different countries, the aim of this study was to determine whether nursing faculty representing diverse regions and cultures would agree on essential core global health competencies. This approach is consistent with the definition of global health that was used to guide the original studies. Global health was viewed as addressing transnational health issues, determinants, and solutions, and differentiated from international health, which focuses on health issues in countries other than one's own, often focused on low or middle income countries. The authors acknowledge that the original list of competencies and the themes that were identified in the qualitative analysis focus more on practice in low resource settings or addressing vulnerable populations, and a list of truly global competencies may also need to include competencies preparing students to practice in high-resource and "high-tech" settings as well. Future research should include respondents from other regions including Asia, Europe, the Middle East, and the Pacific, and should examine differences in which competencies are considered essential among nursing faculty in different regions.

## References

[1] UN System Task Team on the Post-2015 UN Development Agenda (2012). Realizing the future we want for all: Report to the
Secretary-General.

[2] Ventura CAA, Mendes IAC, Wilson L, deGodoy S, Tamy-Maury I, Zarate R (2014). Global health competencies from the perspective of nursing faculty
from Brazilian Higher Education Institutions Rev. Latino-Am. Enfermagem.

[3] Wilson L, Harper DC, Tami I, Zarate R, Salas S, Farley J (2012). Global health competencies for nurses in the Americas. J Prof Nurses.

[4] Koplan JP, Bond TC, Merson MH, Reddy KS, Rodriguez MH, Sewankambo NK (2009). Towards a common definition of global health. Lancet.

[5] Wilson L, Pena LM, Tami-Maury I, Ventura CAA, Warren N, Grajales RAZ (2014). Identifying global health competencies for undergraduate nursing students in
the Americas and in Africa - Presentation.

[6] Harkness JA, Schoua-Glusberg A, Harkness JA (1887). Questionnaires in translation. Cross cultural survey equivalence.

[7] van deVijver F, Leung K (1997). Methods and data analysis for cross-cultural research.

[8] Sandelowski M (2000). Whatever happened to qualitative description. Res Nurs Health.

[9] Sandelowski M, Voils CI, Knafl G (2009). On quantitizing. J Mixed Methods Res.

[10] Polit DE, Beck CT (2008). Nursing research: Generating and assessing evidence for nursing practice, 8th
edition.

[11] Waite R, Nardi D, Killian P (2014). Examinations of cultural knowledge and provider sensitivity in nurse
managed health centers. J Cult Diversity.

[12] American Association of Colleges of Nursing (2008). The essentials of baccalaureate education for professional nursing
practice.

[13] International Council of Nurses (2013). Cultural and linguistic competence.

[14] Rodenborg NA, Boisen LA (2013). Aversive racism and intergroup contact theories Cultural competence in
a segregated world. J Soc Work Educ.

[15] Koshinen L, Tossavainen K (2004). Study abroad as a process of learning intercultural competence in
nursing. Int J Nurs Pract.

[16] World Health Organization (WHO) (2008). World Health Report: Primary health care: Now more than ever.

[17] World Health Organization (WHO) (2014). Fact sheet on universal health coverage.

[18] Frenk J, Chen L, Bhutta AZ, Cohen J, Crisp N, Evans T (2010). Health professionals for a new century transforming education to
strengthen health systems in an interdependent world. Lancet.

[19] World Health Organization (2010). Framework for action on interprofessional education & collaborative
practice.

[20] Hayward LA, Charrette AL (2012). Integrating cultural competence and core values an international
service-learning model. J Phys Ther Educ.

[21] Pacevicius J (2008). Social-psychological competence of leaders structure, empirical
assessment and formative ways. Soc Res.

[22] Foster H. (2015). 4 reasons to add a foreign language to your nursing transcript.

[23] Gebbie KM (2004). Competency to curriculum toolkit: Developing Curricula For Public Health
Workers.

[24] Smith D (2005). Organizing for disaster preparedness. J Commun Pract.

[25] Association of Schools of Public Health (2011). Global health competency model final version 1.1.

